# Direct versus Indirect Techniques to Menage Uncomplicated Crown Fractures of Anterior Teeth Following Dentoalveolar Trauma

**DOI:** 10.3390/dj9020013

**Published:** 2021-01-20

**Authors:** Roberto Apponi, Alberto Murri dello Diago, Vittorio Colombini, Giorgia Melis

**Affiliations:** 1 Department of Dentistry and Oral Maxillofacial Surgery, University of Modena and Reggio Emilia, 41124 Modena, Italy; alberto.murridellodiago@gmail.com (A.M.d.D.); vittorio.colombini@hotmail.it (V.C.); 2 Department of Surgical Sciences, Dental Prosthetic Division, University of Cagliari, 09121 Cagliari, Italy; giorgiamel51@gmail.com

**Keywords:** trauma, uncomplicated crown fracture, direct composite restorations, veneers, indirect anterior restorations

## Abstract

Dental trauma are the most common reasons for dental fractures in the anterior area, they have an incidence of 5% in the population, and in permanent teeth, they are mainly caused by sports. The most involved teeth are the maxillary anterior teeth. Direct composite restorations and indirect ceramic restorations are the therapy of choice for restoring anterior teeth after fracture when is not possible to reattach the tooth fragment. The treatment options in uncomplicated coronal fractures depend on various factors such as the amount of residual dentinal enamel tissue, the relationship with the gingival profiles, and the age of the patient. The purpose of this article is to discuss the option of using direct or indirect restorative techniques in the treatment of traumatically fractured anterior teeth and to analyze the advantages and disadvantages of the two methods.

## 1. Introduction

Dental trauma (DTI) is an impact injury to the teeth and/or other hard and soft tissues in and around the mouth and oral cavity [1]. The consequences for the individual are not only physical or economic but have an impact on the patient’s quality of life [2]. The incidence of dental trauma affects about 5% of the population on a global scale, and the prevalence shows a wide range from 6% to 59% with a large variation depending on the geographical area [3]. The risk of suffering dental trauma has been reported to depend on several factors such as age, circumstances, geographic location, behavior, and culture, and is increasing in the home, school, and sports environment [3]. Despite these variations, there is a general trend indicating that a third of all preschool children (primary teeth) and a quarter of adolescents and adults (permanent teeth) have suffered dental trauma at least once in their lifetime. In permanent dentition, sports activities are the number one cause of trauma [3]. The nature of sport is a source of variation due to the amount of physical contact and popularity. Other causes of dental trauma reported in studies are collisions, assaults, bicycle accidents, and motor vehicle accidents [4]. Most dental trauma in primary and permanent dentition involves the front teeth. The upper central and lateral incisors are most commonly subject to trauma teeth. In most cases, the trauma concerns the single tooth, but some events, especially sports, are more likely to involve multiple teeth [4]. Many studies have shown that males suffer trauma more frequently (1.3–2.5 times) than females in permanent dentition, probably due to a greater propensity for contact sports, violent behavior, and a lower maturity than females [4]. Age is another risk factor. Studies indicate that dental trauma is more common in the younger population (toddlers, children, adolescents, and young adults) due to increased physical and sporting activity [4]. The International Association of Dental Traumatology (IADT) has developed a classification of traumatic tooth injuries [5,6] and developed guidelines to provide dentists and patients with the best treatment choices [5]. Patients with dental trauma should be aware that some injuries require multiple appointments and checkups over time. Success rates can vary as much as the nature and circumstances causing the trauma. In carrying out therapeutic procedures, several aspects must be evaluated. Management of dental trauma is complex and can be more complex in growing patients. In fact, poor cooperation or the fear of feeling pain during operative procedures is an evidence to keep in mind. For this reason, even anesthetic techniques, when necessary, can help to improve the approach to therapies [7]. Following a dental trauma, the resulting aesthetic defect of the anterior teeth can be significant. Complicated or uncomplicated coronal fractures can be restored through different options. When possible, the reattachment of tooth fragment is the first choice. Using correctly adhesive protocols, restoration will be performed restoring function and esthetic appearance [8]. If fragment reattachment is impossible, direct or indirect restorations are the alternatives. Regarding direct and indirect restorations, the clinician must choose which of the two methods to use. In literature, there are no precise guidelines regarding this choice. Composites, infiltrating resins, ceramics, layering techniques, and CAD-CAM techniques can simplify the therapy and make it predictable [9,10]. Following any dental trauma, the pulp can undergo complications such as necrosis, root canal calcification (PCO), infection of the root canal system, or internal resorption. Similarly, the root portion and peri-root tissues can suffer severe damage including apical periodontitis, external resorption (inflammatory resorption or replacement resorption), disorders in root development, loss of periodontal ligament (ankylosis), soft tissue recession, and/or fibrous healing [11]. The literature provides consolidated guidelines in the case in which endodontic treatment is necessary even if, in some cases, especially after reimplantation, there are still some uncertainties [12]. It is estimated that 26–76% of lesions cause permanent tooth loss [13]. In this case, therapies in growing patient are autotransplantation, closure of spaces by orthodontic treatment and prosthetic, or implant-prosthetic rehabilitation with mini-implants [14,15]. Other complications include misalignment, discoloration of the teeth, difficulty in eating, esthetics compromised, and discomfort. It is known in the literature that orthodontic treatments exist in case of misalignments, intrusion of the traumatized tooth, discoloration, and compromised esthetics aimed at promoting correct prosthetic restoration [16,17]. The prognosis of lesions depends on immediate and correct management and is reduced in case of simultaneous lesions on the same teeth. Wang et al. also reported that unrestored lesions showed an almost threefold increase in the probability of pulpal necrosis compared to treated teeth [18]. The treatment of uncomplicated coronal fractures is essential not only from an aesthetic but also from a prognostic point of view. The purpose of this article is to discuss the option of using direct or indirect restorative techniques and to evaluate the selection criteria as in the two cases presented as an example of techniques after uncomplicated crown fracture.

## 2. Methodology

During the emergency visit, it is necessary to collect a correct dental history and on the physical examination following the IADT guidelines [5], it is necessary to clinically observe what the patient reported in the medical history. Following the execution of a periapical radiograph to verify that there are no periradicular reactions and there are no signs of root fractures and having ascertained that there are no fractures of the alveolar process, it is necessary to perform a pulp sensitivity test to verify the vitality of the tooth.

### 2.1. Direct Restoration Technique

**Figure 1:**
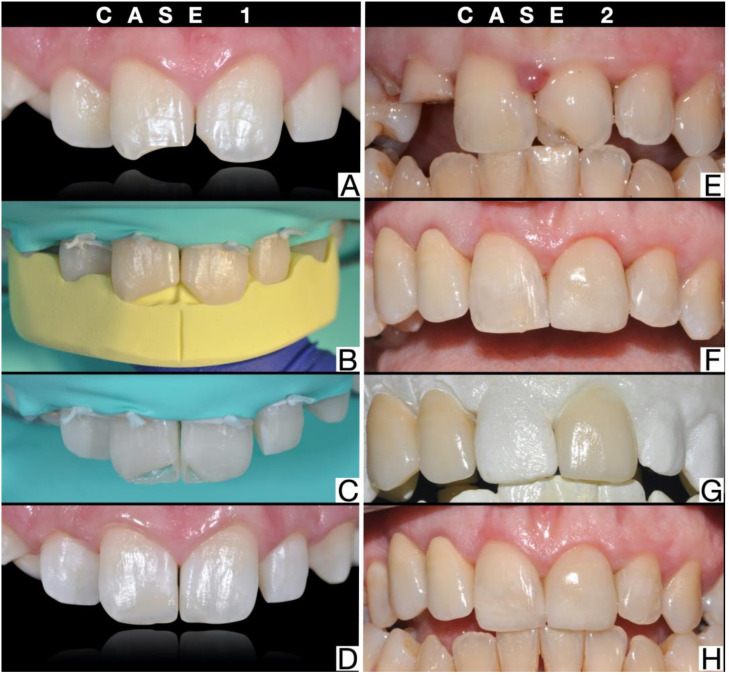
Case 1: (**A**) image after trauma of 1.1 and 2.1, (**B**) silicone guide, (**C**) first palatal increment, and (**D**) final restoration. Case 2: (**E**) image after trauma of 2.1, 1.2, and 1.3; (**F**) provisional resin crow; (**G**) prototyped models; and (**H**) final restoration.

In the first case, direct reconstructive techniques are described, following uncomplicated crown fractures of 1.1 and 2.1. Teeth 1.1 and 21 have no root fractures, no mobility, and have maintained pulp vitality (Figure 1A). The technician makes a silicone guide necessary for the restoration of the fractured tooth (Figure 1B). Rubber dam isolation is essential, must be wide, and extends from premolar to premolar for the anterior sector [19]. On the vestibular and palatal enamel, a functional bevel must be performed to camouflage the transition area between restoration and enamel and to remove unsupported enamel prisms. It then proceeds with the conventional steps of etching, application of the primer, and bonding. Once the silicone guide has been positioned, the first increment is made palatally with enamel composite resin with a thickness of about 0.5 mm (Figure 1C) [20]. The transparent anatomical matrices are positioned interproximally and the interproximal surfaces, incisal margins, and the dentin body are layered with composite resin. In the vestibular empty space between the mamelons and the incisal margin, the opalescent masses are applied if there is opalescence in the remaining part of the fractured tooth or in the contralateral tooth. A final increment of enamel composite (no thicker than 0.5 mm) is applied to the buccal surface [21]. Restored tooth can now be finished using fine-grained diamond finishing burs (30–40 μm). For the polishing steps, it is possible to use medium and fine-grained finishing discs to provide the surface gloss of the natural enamel and goat hair/suede/felt brushes that allow to develop a high gloss. Finishing abrasive strips can be used to remove excess interproximal composite and refine emergence profiles (Figure 1D) [22].

### 2.2. Indirect Restoration Technique

In the second case, indirect reconstructive techniques are described, following multiple crown fractures, with particular attention to the indirect reconstructive technique of tooth 2.1 after uncomplicated crown fracture. Tooth 21 had no root fractures, no mobility, and had maintained pulp vitality (Figure 1E). The technician creates a diagnostic wax-up of the traumatized tooth on the model and a silicone key to print the mock-up. During the second appointment, the clinician prints the mock-up with the silicone key in the patient’s mouth and evaluates the esthetic result together with the patient. Through the mock-up, the clinician prepares the tooth with diamond burs to create a minimally invasive indirect adhesive restoration in order to preserve as much enamel as possible for better adhesion [23]. Once the preparation of the tooth is completed, an impression is taken with a digital scanner or with elastomeric materials such as polyvinylsiloxane or polyether. The clinician will then reprint the mock-up on the prepared tooth as a temporary restoration (Figure 1F). The technician will receive the impressions and make the veneer (Figure 1G). During the third appointment, the clinician removes the provisional mock-up and tries-in the restoration in the patient’s mouth. The cementation phases are carried out with adhesive techniques that differ according to the type of ceramic used [24]. The marginal areas are finished with silicone tips and the finishing abrasive strips are used to remove excess interproximal composite (Figure 1H).

## 3. Discussion

In case of anterior teeth fracture as a result of a trauma, it is possible to use direct and indirect techniques to restore the correct morphology of the teeth concerned. Several choices of materials for both techniques are available. Both techniques have positive and negative aspects that will lead the dentist to make choices [25]. Composite resins, mainly used in direct procedures, can be indicated for both direct and indirect techniques and their widespread use derives from their acceptable longevity at relatively low costs [26]. On the other hand, ceramic materials such as lithium disilicate, feldspathic ceramic and zirconia are used exclusively for indirect techniques. Although the reliability of composite materials is known, many clinicians still resort to more invasive indirect procedures, relying on their technicians to create veneers or crowns with optical properties similar to those of natural teeth. In fact, there are no absolute choice criteria between the two techniques; therefore, the factors to consider are the invasiveness, the cost, and the longevity of the restoration. Among the advantages of direct techniques, it is known that they have a few endodontic complications, present the possibility of reintervening in the event of a further fracture, and allow immediate restoration of aesthetics in a single appointment. An additional benefit of direct restorations is the preservation of all future treatment options. Compared to indirect techniques, however, they have lower strength, greater surface roughness and polymerization shrinkage. The indirect ceramic techniques, on the other hand, provide a better aesthetic appearance because they have translucent characteristics similar to those of the enamel and advantages in terms of color, rigidity, marginal compatibility, and microinfiltrations [27]. The technical sensitivity, timing, repairability, and economic condition of the patient must be considered when choosing between direct and indirect restorations. In view of the high incidence of dentoalveolar trauma recorded during sports activities, it is clear that adopting correct prevention strategies is essential especially in those patients who have already suffered a trauma. It is known that the use of mouthguards has been extensively studied and represents one of the most effective forms of prevention during sports activities [28,29]. In addition, the knowledge of techniques, tissue healing processes, and the ability to manage long-term treatments determine the success of the therapy [29,30].

## 4. Conclusions

Recently, with the improvement of dental technologies, dentists can provide more conservative restorations, keeping the integrity of the tooth at an optimal level. In the two cases presented as an example of techniques, both methods can be recommended for the coronal recovery of traumatized anterior teeth. There are no strict selection criteria but different assessments will lead the clinician to opt for one or the other technique. Age certainly plays a fundamental role in the choice and direct restorations are preferred in growing or young patients as chosen in the second case and indirect restorations in more complex cases as described in the first case. Even the propensity of the individual dentist can affect the setting of the treatment plan. Prevention plays an important role and especially in sports is recommended to use all available aids such as mouthguard.

## Data Availability

The data presented in this study are available in this article.
